# Impact of Sex Hormones on Macrophage Responses to *Coxiella burnetii*

**DOI:** 10.3389/fimmu.2021.705088

**Published:** 2021-12-20

**Authors:** Laetitia Gay, Cléa Melenotte, Alexandre Lopez, Benoit Desnues, Didier Raoult, Marc Leone, Soraya Mezouar, Jean-Louis Mege

**Affiliations:** ^1^ Aix-Marseille University, Institut de Recherche pour le Développement (IRD), Assistance Publique - Hôpitaux de Marseille (APHM), Microbes, Evolution, Phylogeny and Infection (MEPHI), Marseille, France; ^2^ Department of Immunology, Institut Hospitalo-Universitaire (IHU)-Méditerranée Infection, Marseille, France; ^3^ Department of Anesthesia and Intensive Care, Hôpital Nord, Aix-Marseille Univ, Assistance Publique - Hôpitaux de Marseille (APHM), Marseille, France; ^4^ Aix-Marseille University, Assistance Publique - Hôpitaux de Marseille (APHM), Hôpital de la Conception, Laboratoire d’Immunologie, Marseille, France

**Keywords:** *Coxiella burnetii*, cytokines, inflammatory response, MDMs, sex hormones

## Abstract

**Introduction:**

Q fever, a zoonosis caused by *Coxiella burnetii*, affects more males than females despite a similar level of exposure. A protective role of estradiol has been reported in mice, suggesting that sex hormones are involved in *C. burnetii* infection. We wondered whether the responses of monocytes and monocyte-derived macrophages (MDMs) to *C. burnetii* are influenced by sex hormones.

**Materials and Methods:**

The bacterial intracellular fate in monocytes was studied using quantitative PCR, and monocyte cytokine production in response to *C. burnetii* was assessed using qRT-PCR and immunoassays. Before infection, MDMs from males and females were incubated with testosterone and estradiol, respectively.

**Results:**

Bacterial uptake and persistence were similar in monocytes from males and females but were slightly increased in male MDMs. The expression of inflammatory genes, including those encoding TNF and CXCL10, was higher in MDMs from females than in MDMs from males infected by *C. burnetii*. Adding testosterone to male MDMs amplified their immunoregulatory properties, including increased expression of *IL10* and *TGFB* genes and TGF-β production in response to *C. burnetii.* In contrast, adding estradiol to MDMs from females had no effect on their inflammatory profile.

**Conclusion:**

The stronger inflammatory profile of macrophages from females may have a protective role, likely under estrogen control, while testosterone may affect disease progression by promoting an anti-inflammatory response. This finding may have consequences for personalized management of patients with Q fever.

## Introduction

Q fever is an infectious disease caused by the intracellular bacterium *Coxiella burnetii*. In humans, primary infection (acute Q fever) may be symptomatic (40%), and the evolution toward persistent focalized infection is observed in only 1-5% of cases, dominated by cardiovascular and osteo-articular manifestations (1). While Q fever seroprevalence is similar between males and females, *C. burnetii* infection is characterized by a dimorphism, with a male/female ratio ~2.2 reported worldwide (2, 3). The levels of anticardiolipin antibodies are higher in males than in females (4). In persistent *C. burnetii* focalized infection, the male/female ratio is 2.6 and 7.5 in endocarditis and vascular infection, respectively, and being male is associated with an increased risk of vascular infection, independently of age (2, 5). It is likely that the male/female dimorphism observed in Q fever is related to sex hormones, as supported by three major clinical observations (6, 7). First, sexual dimorphism is not reported before puberty; second, *C. burnetii* infection induces complications during pregnancy in which variations in sex hormones are prominent and; third, the increased risk of cardiovascular infection is probably under hormonal influence (2, 8).

The effect of female sex hormones on *C. burnetii* persistence has been evoked by different reports. Adding progesterone to JEG-3 cells inhibits *C. burnetii*-containing vacuole development and bacterial persistence (9). Surprisingly, more than 86% of genes are differently modulated in male and female mice infected by *C. burnetii* for 24 hours, and 60% of this gene modulation is due to sex hormones, as demonstrated by gonadectomy of males and females. Modulated genes are associated with the inflammatory response in males, but not in females (10). It has also been shown that β-estradiol plays a protective role and controls tissue infection and granulomatous response in mice (11).

Monocytes and macrophages play a major role in the antibacterial response. Following a bacterial infection, macrophages generally mount an inflammatory program (M1 program) with up-regulation of genes encoding cytokines such as tumor necrosis factor (TNF), interleukin (IL)-6, IL-1, IL-12, IL-23, chemokines such as CXCL10 and surface receptors such as CCR7 (12). However, *C. burnetii* infection leads to an immunoregulatory profile (M2 program) in human macrophages. Indeed, in *C. burnetii*-infected macrophages, genes encoding immunoregulatory cytokines such as IL-10 and transforming growth factor (TGF)-β1 and cytokine receptors such as the IL-1 receptor antagonist (IL-1RA) are up-regulated, and this M2 environment promotes *C. burnetii* survival (12–15).

Since monocytes and macrophages are the major targets of *C. burnetii* (16), we studied the uptake and persistence of *C. burnetii* in monocytes and monocyte-derived macrophages (MDMs) from healthy males and females. *C. burnetii* uptake and persistence was similar in monocytes from males and females but was slightly increased in MDMs from males. We also found that female MDMs infected by *C. burnetii* had an inflammatory profile greater than that of male MDMs. Interestingly, the anti-inflammatory profile of testosterone-pretreated MDMs from males seemed to be associated with defective elimination of *C. burnetii*.

## Materials and Methods

### Bacteria

*Coxiella burnetii* organisms in phase I (Nine Mile strain, RSA493) were cultured in L929 cells for 10 days (17). Briefly, sonicated cells were centrifuged, and bacterial pellets were centrifuged at 10,000 × *g* for 10 minutes, then washed and stored at -80°C. The concentration of organisms was determined by Gimenez staining, and bacterial viability was assessed using the LIVE/DEAD BacLight bacterial viability kit (Molecular Probes, Eugene, OR, USA).

### Human Monocytes and MDMs

Blood samples (leucopacks) that we used in our study come from the French Blood Establishment (Etablissement français du sang, EFS) that carries out donor inclusions, informed consent and sample collection. Through a convention established between our laboratory and the EFS (N°7828), buffy coats were obtained from healthy blood donors. Peripheral blood mononuclear cells (PBMCs) were isolated after centrifugation on Ficoll cushions and cultured in RPMI 1640 containing 10% of heat-inactivated fetal bovine serum (FBS), 2 mM L-glutamine, 100 U/mL penicillin and 50 µg/mL streptomycin (Life Technologies, Carlsbad, CA, USA) for 2 hours. After washing, adherent cells were recovered and designated as monocytes because more than 95% of the cells expressed CD14, a monocyte marker, as assessed by flow cytometry. Monocytes were then differentiated into macrophages by incubating them in RPMI 1640 containing 10% of heat-inactivated human AB serum (MP Biomedicals, Solon, OH, USA), 2 mM glutamine and antibiotics for 3 days and in RPMI 1640 supplemented with 10% of heat-inactivated FBS for 4 additional days **(** **Supplemental Figure 1**) (18). More than 95% of the differentiated cells were MDMs, as determined by the expression of CD68. In some experiments, MDMs from males and females were pre-treated for 24 hours with 1 nM estradiol E2 and 1 nM testosterone (Tocris Bioscience, Bristol, United Kingdom), respectively, before *C. burnetii* stimulation (**Supplemental Figure 1**). We performed a dose test with the following concentrations: 0, 1, 10, 100, and 1000 nM. We observed that the 1 nM testosterone concentration resulted in the highest IL-10 secretion in macrophages and the 1 nM estradiol concentration resulted in the highest TFN-α secretion (**Supplemental Figure 2**) as previously reported (19).

### Antimicrobial Activity of Monocytes and MDMs

Monocytes and MDMs (3×10^5^ cells/assay) were incubated with living *C. burnetii* organisms (bacterium-to-cell ratio of 50:1) in 24-well dishes for 4 hours. After extensive washing to remove free bacteria (time designed as day 0), infected cells were cultured for 9 additional days. DNA (50 µL volume) was extracted from the total infected cells/assay every 3 days using DNA Mini Kit (Qiagen, Courtaboeuf, France). Infection was quantified using 2 µL of DNA and real time quantitative PCR (qPCR) performed with specific primers targeting the *C. burnetii com1* gene (17). Bacterial uptake was expressed as the number of bacterial DNA copies within monocytes and MDMs. The results of their intracellular fate were expressed as percentage of persistence (number of bacterial DNA copies at days 3, 6 and 9 compared to that at day 0).

### RNA Isolation and qRT-PCR

Total RNA was extracted from MDMs (1×10^6^ cells/assay) using the RNeasy Mini Kit (Qiagen) and DNase I treatment and evaluated using a spectrophotometer (Nanodrop Technologies) (17).

RT-PCR was performed using a Moloney murine leukemia virus-reverse transcriptase kit (Life Technologies) and oligo(dT) primers, Smart SYBRGreen fast Master kit (Roche Diagnostics, Basel, Switzerland), 5 µl DNA and a CFX Touch Detection System (Bio-Rad, Marnes-la-Coquette, France) using specific primers listed in **Supplementary Table 1**. The results were normalized using the housekeeping *ACTB* gene encoding β-actin and were expressed as fold change using the following formula: fold change=2^−ΔΔCt^, where ΔΔCt=(Ct_Target_ - Ct_Actin_)_stimulated/infected_ - (Ct_Target_ - Ct_Actin_)_non-stimulated/infected_ (18). The threshold cycle (Ct) was defined as the number of cycles required to detect the fluorescent signal. The expression of genes was considered modulated when fold change was ≥ 1.5.

### Immunoassays

The release of TNF, IL-10 and TGF-β1 was quantified in cell supernatants using specific immunoassay kits provided by R&D Systems (Bio-Techne, Novel Châtillon-sur-Seiche, France). The sensitivity of assays was 5.5 pg/mL for TNF, 3.9 pg/mL for IL-10 and 3.4 pg/mL for TGF-β1.

### Statistical Analysis

Statistical analysis was performed using Prism 7.0 (Graphpad Software Inc.). Data were compared using non-parametric (Mann-Whitney *U* test) statistical tests. Correlation tests were performed with a Pearson test or a non-parametric Spearman test and represented graphically by a regression line. Differences were considered significant when *p*<0.05.

## Results

### Antimicrobial Activity of Male and Female Monocytes and MDMs

**Figure 1:**
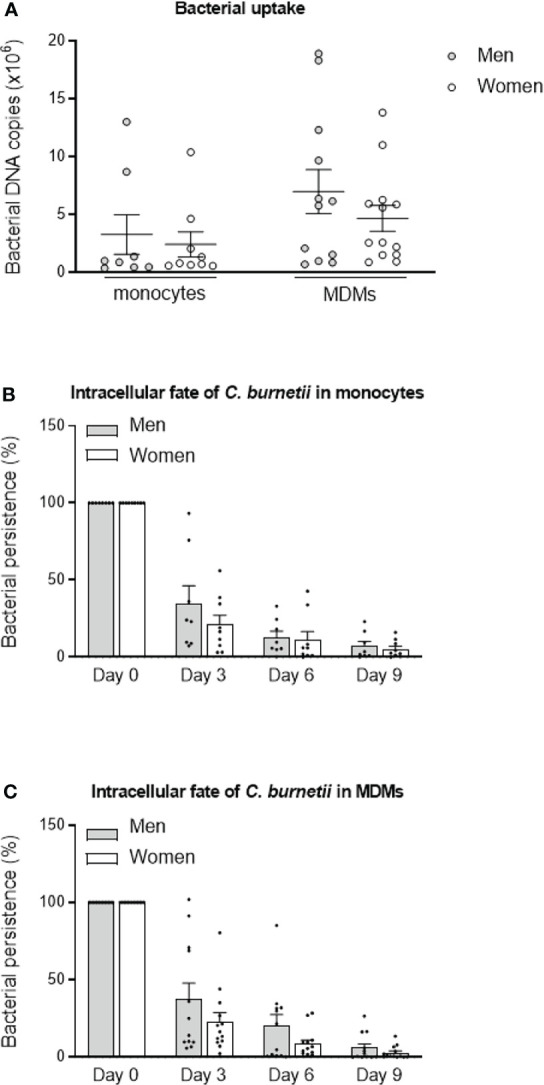
C. *burnetii* uptake and persistence. **(A)** Monocytes from healthy donors from males (n=8) and females (n=9) and MDMs (12 males and 13 females) were infected with *C. burnetii* (50 bacteria per cell) for 4 hours. The number of bacterial DNA copies within cells was determined by qPCR. The tests were performed in duplicate for each individual. After the bacterial uptake phase (designated day 0), **(B)** monocytes and **(C)** MDMs were cultivated for 9 days, and the presence of bacterial DNA copies was assessed every 3 days. The bacterial fate at day 3, 6 and 9 was calculated in percentage relative to day 0. Data for men (in grey) and female (in white) represent mean ± standard error of the mean (duplicate was performed for each individual). Statistical analyses were performed using Mann-Whitney *U* test (men *vs.* women).

The uptake of *C. burnetii* organisms (50 bacteria per cell) by monocytes and MDMs was evaluated by qPCR. The bacterial uptake is the same for monocytes from males (n=8) and females (n=9) (*p=0.8148*). Similar observations were observed for MDMs, with a tendency to increase of the bacterial DNA copies in cells from males (7 × 10^6^, n=12) compared to females (4.7 × 10^6^, n=13) but without significance (*p=0.6214*) (**Figure 1A**).

We then studied the intracellular fate of *C. burnetii* in monocytes and MDMs. No significant differences of bacterial persistence were observed at day 3, 6 and 9 (*p=0.5554*, *p=0.6730* and *p=0.8884*, respectively) between monocytes from males (n=8) and females (n=9). Of note, bacterial persistence tended to be higher in monocytes from males (34.8%) than from females (21.1%) at day 3, but similar levels were observed after 6 and 9 days (**Figure 1B**). The persistence of *C. burnetii* also seemed higher in MDMs from males (n=12) than from females (n=13) at day 3 (37.5% *vs.* 23% for males and females, respectively, *p=0.6495*), day 6 (20.3 *vs.* 8.6% for males and females, respectively, *p=0.6590*) and day 9 (6.0 *vs.* 2.8% for males and females, respectively, *p=0.8618*) (**Figure 1C**). Since inter-donor difference may be a bias, we performed the same experiments with monocytes and MDMs from the same donors. As shown in the **Supplemental Figure 3**, no differences were observed between cells from males and females whether for bacterial uptake or persistence. These results reported that bacterial uptake and persistence were similar in monocytes and macrophages from males than in those from females and highlighted a heterogeneous response from donors of both sexes.

### Transcriptional Profile of *C. burnetii*-Infected Male and Female MDMs

**Figure 2:**
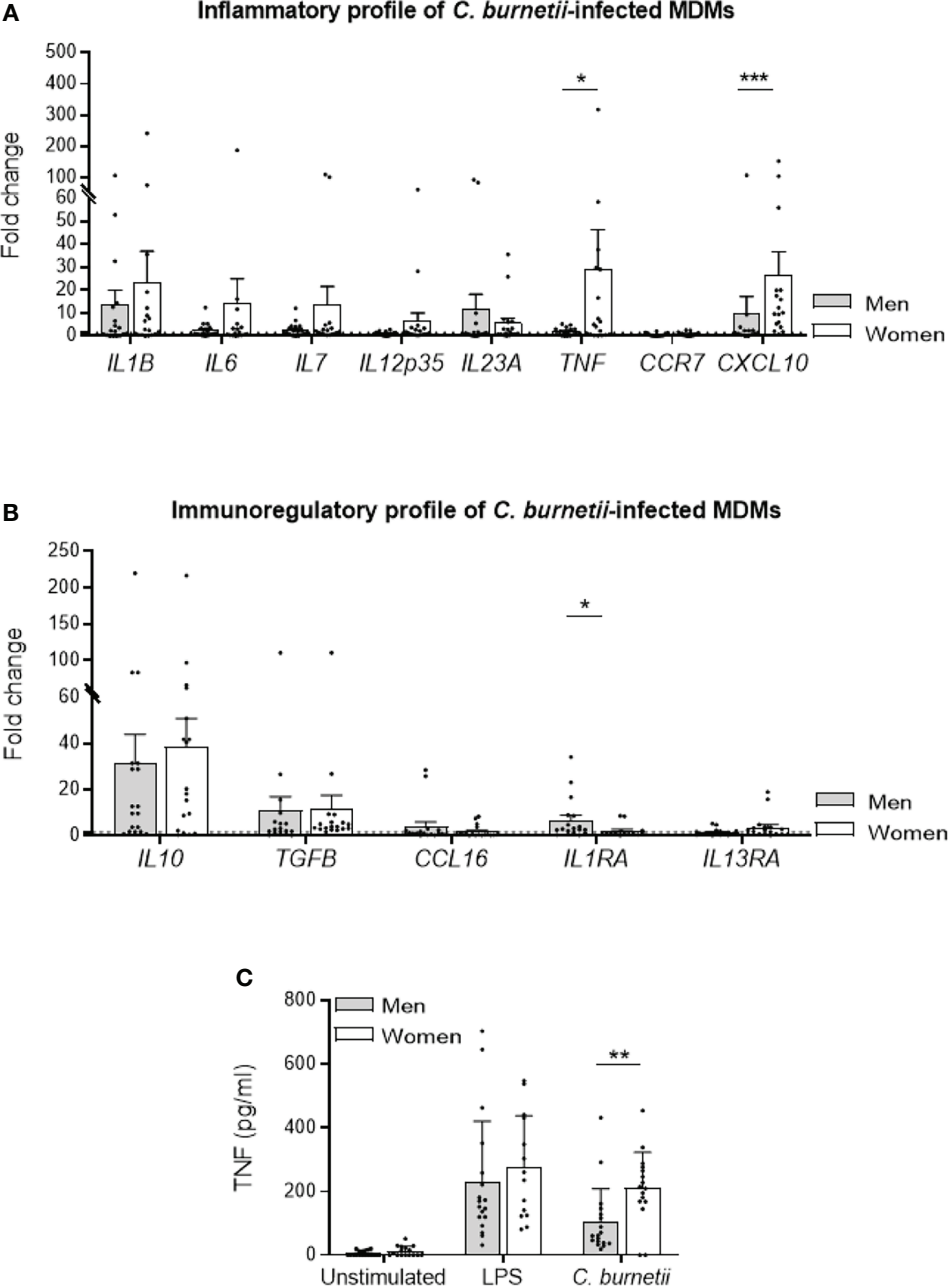
M_1_/M_2_ polarization profile of *C. burnetii*-infected MDMs. MDMs from healthy donors from males (n=18) and females (n=18) were infected with *C. burnetii* (50 bacteria per cell) for 6 hours and transcriptional responses were assessed by qRT-PCR and constant amount of total DNA. The expression of MDM **(A)** M_1_ and **(B)** M_2_ polarization genes was investigated by qRT-PCR and expressed as fold change relative to unstimulated cells was calculated. Gene expression was considered modulated when the fold change was ≥ 1.5 (indicated by the dotted line). **(C)** The release of TNF by MDMs, infected or not by *C. burnetii* (50 bacteria per cell) or stimulated with 1 µg/mL LPS for 24 hours, was determined by immunoassays. Data for men (in grey) and female (in white) represent mean ± standard error of the mean (duplicate was performed for each individual). Statistical analyses were performed using Mann-Whitney *U* test (men *vs.* women). ^*^ *p ≤* 0.05, ^**^ *p ≤*0.01 and ^***^ *p ≤* 0.001.

Because the elimination of *C. burnetii* by human MDMs is closely related to their inflammatory state (16), we investigated the inflammatory profile of MDMs from males (n=18) and females (n=18) infected with *C. burnetii* (50 bacteria per cell) for 6 hours using qRT-PCR and constant amount of total DNA. Among the 8 M1 genes investigated, 3/8 genes were up-regulated (fold change > 1.5) in MDMs from males (*IL1B*, *IL23A* and *CXCL10*), and 7/8 (*IL1B*, *IL6*, *IL7*, *IL12p35*, *IL23A*, *TNF* and *CXCL10*) for females. The expression of genes encoding *CXCL10* and *TNF* was significantly higher (*p=0.0008* and *p=0.041*, respectively) in MDMs from females than in those from males (**Figure 2A**). We also analyzed the expression of 5 genes considered as M2 genes. Clearly, the *IL10* and *TGFB* genes were largely up-regulated in response to *C. burnetii*, but their expression was similar in MDMs from males and females. Only, *IL1RA* gene expression was significantly (*p=0.016*) up-regulated only in MDMs from males compared to those of females (**Figure 2B**).

Next, we investigated TNF release by MDMs from donors of both sexes infected by *C. burnetii* (50 bacteria per cell) or stimulated by LPS, a potent agonist of TNF production, for 24 hours. While TNF release was similar in LPS-stimulated in MDMs from males and females, the infection by *C. burnetii* lead to a significant decreased of cytokine level in MDMs from males compared to females (mean 103 *vs.* 211 pg/mL, respectively, *p=0.004*) (**Figure 2C**). Taken together, these results showed that *C. burnetii* induced a more pronounced inflammatory profile in MDMs from females in which TNF occupied a prominent position.

### Role of Sex Hormones in *C. burnetii* Infection

**Figure 3:**
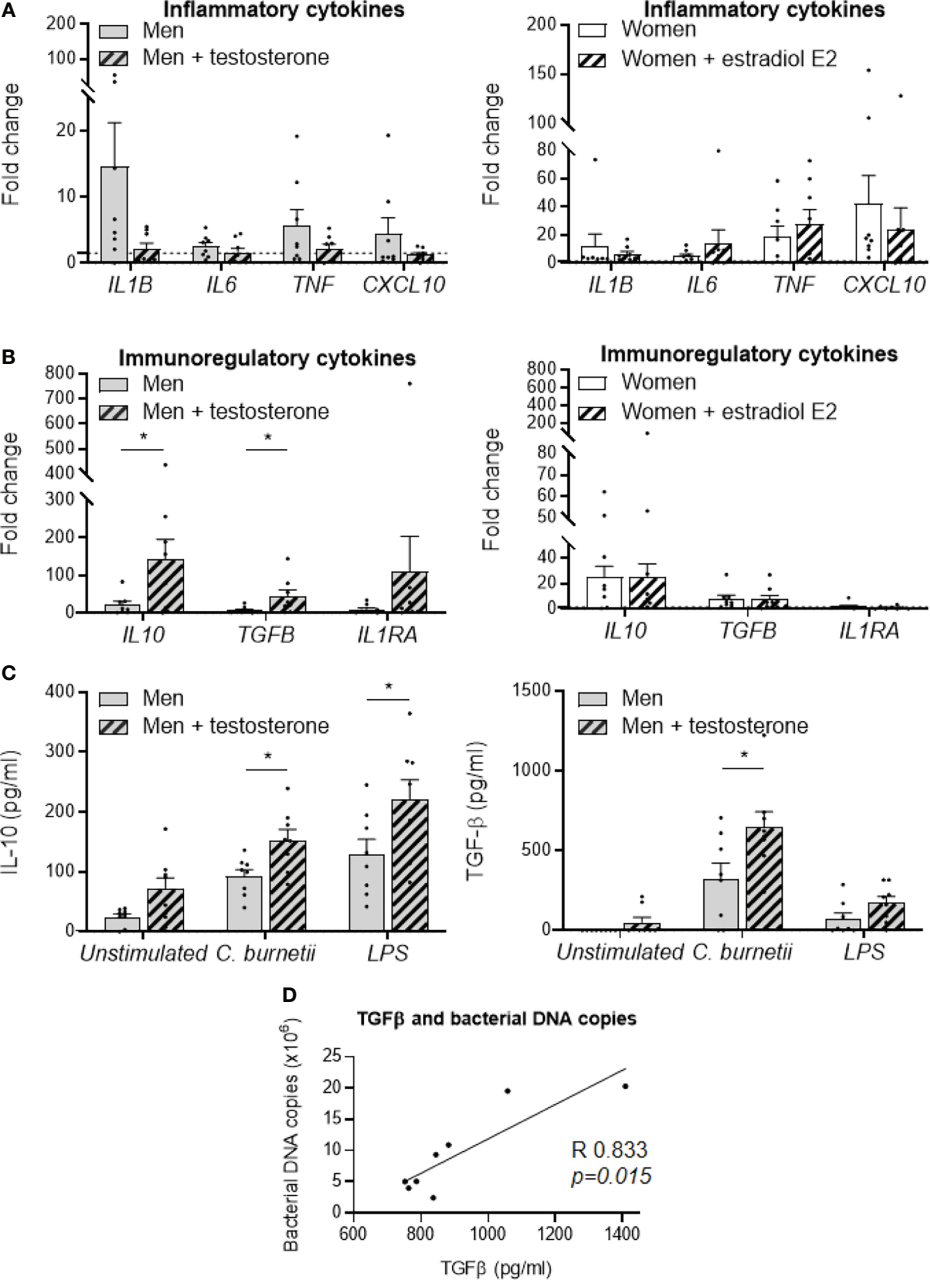
Role of sex hormones in MDM responses to *C. burnetii.* MDMs from healthy donors from males (n=8) and females (n=8) were treated with testosterone and estradiol E2, respectively, for 24 hours before *C. burnetii* (50 bacteria per cell) stimulation. The expression of MDM **(A)** M_1_ and **(B)** M_2_ polarization genes was investigated by qRT-PCR, constant amount of total DNA and expressed as fold change relative to unstimulated cells was calculated. Gene expression was considered modulated when the fold change was ≥ 1.5 (indicated by the dotted line). Left panels concern MDMs from males +/- treated with testosterone and the right panels concern MDMs from females +/- treated with estradiol E2. **(C)** The release of IL-10 and TGF-β by MDMs from males (n=8) and females (n=8) infected by *C. burnetii* (50 bacteria per cell) or stimulated with 1 µg/mL LPS for 24 hours was determined by immunoassays. Data for men (in grey) and female (in white), with (striped bar) or without pre-treatment, represent mean ± standard error of the mean (duplicate was performed for each individual). Statistical analyses were performed using Mann-Whitney *U* test (men *vs.* women). ^*^ *p ≤* 0.05. **(D)** Correlation between the levels of released TGF-β and the number of *C. burnetii* DNA copies uptake by MDMs from males after 24 h. Statistical analysis was performed using Spearman test.

In line with the previous results, we next investigated the effect of sex hormones on MDM response to *C. burnetii*. For that purpose, MDMs from males (n=8) and females (n=8) were treated with 1 nM of testosterone and 1 nM estradiol E2, respectively, for 24 hours before stimulation with *C. burnetii* (50 bacteria per cell). Testosterone decreased the expression of *IL1B* gene, with a fold change value reduced from 14 to 2, and down-regulated that of *TNF* and *CXCL10* genes, with a fold change reduced from 4 to less than 2, but no significant differences were observed (**Figure 3A**, left panel). In contrast, estradiol E2 did not induce neither expression modulation nor significant difference of M1 investigated genes in MDMs from females (**Figure 3A**, right panel). We also studied the effect of testosterone and estradiol E2 on the expression of M2 genes. Testosterone dramatically increased the expression of *IL10* and *TGFB* genes (*p=0.045* and *p=0.0207*, respectively) in MDMs from males, and that of *IL1RA* gene to a lesser extent but without significance (*p=0.3823*) (**Figure 3B**, left panel). In contrast, estradiol E2 did not affect the expression of the genes encoding *IL10*, *TGFB* and *IL1RA* by MDMs from females (**Figure 3B**, right panel). As testosterone greatly amplified the transcription of immunoregulatory genes induced by *C. burnetii*, we wondered whether this effect was specific. We found that the expression of the *IL10* gene was increased in MDMs from males (n=8) pre-treated with testosterone and stimulated by LPS, with a fold change of 60 *vs*. 30 without testosterone pretreatment (*p=0.026*). However, testosterone was unable to modulate the expression of *TGFB* and *IL1RA* genes (*p=0.1847* and *p=0.4811*, respectively) (**Supplemental Figure 4**). As the variation in the fold change induced by testosterone was also greater when MDMs from males were infected by *C. burnetii* (150 *vs.* 20), it is likely that the effect of testosterone on gene expression was largely dependent on *C. burnetii*.

To confirm our findings, MDMs from female donors were treated with testosterone and MDMs from male donors with estradiol. As shown in the **Supplemental Figure 5**, we observed that estradiol treatment results in higher expression of pro-inflammatory genes *TNF* and *IL1B* in *C. burnetii*-stimulated MDMs from male and female donors, but with a more pronounced effect in MDMs from female donors. On the other hand, testosterone treatment resulted in significantly higher *IL10* gene expression only in *C. burnetii*-stimulated MDMs from male donors.

Next, we studied the effect of testosterone on the release of immunoregulatory cytokines induced by *C. burnetii* or LPS. Testosterone pre-treatment of MDMs from males infected by *C. burnetii* or stimulated by LPS significantly increased IL-10 release (150 *vs.* 90 pg/mL, *p=0.013* and 220 *vs.* 130 pg/mL, *p=0.045*, respectively) (**Figure 3C**, left panel). Note that testosterone pre-treatment also did not affect the release of IL-10 by non-infected MDMs (70 *vs.* 25 pg/mL, *p=0.10*). In contrast, testosterone had only a marginal role in the release of TGF-β by unstimulated or LPS-stimulated MDMs from males, but it significantly increased the release of TGF-β induced by *C. burnetii* infection (640 *vs.* 320 pg/mL, *p=0.036*) (**Figure 3C**, right panel). Interestingly, we found that this specific release of TGF-β induced by *C*. *burnetii* infection of MDMs from males was correlated with the number of *C. burnetii* DNA copies uptake by MDMs from males after 24 h (*p=0.015*, Spearman r=0.833) (**Figure 3D**).

## Discussion

As epidemiological studies and animal models have suggested that the host defense against *C. burnetii* is different in males and females, we wondered if such sexual dimorphism is observed in human myeloid cells, the natural targets of *C. burnetii*. We first showed that sex did not affect the uptake of *C. burnetii* by human monocytes and macrophages. Few studies have investigated the impact of sex differences on phagocytosis. Toxicology models of ethanol intoxication show increased phagocytosis in neutrophils from female rats as compared to males (20). Peritoneal macrophages from female mice internalize more zymosan particles than those of males. In ovariectomized mice, zymosan phagocytosis is significantly reduced compared to female controls (21), suggesting that sex hormones play a major role in zymosan internalization. The phagocytic capacity of rat peritoneal macrophages is increased by estradiol E2 or progesterone treatment (22). Testosterone increases phagocytosis by rat peritoneal macrophages in a dose-dependent manner, but it has also been shown that testosterone does not affect the phagocytic activity of human monocytes (22–24). It is likely that uptake mechanisms are differently affected by sex hormones in human and murine macrophages.

The microbicidal activity against *C. burnetii* of monocytes and MDMs from females was more efficient than those from males. It is likely that this trend reflects a sex-biased production of cytokines. *C. burnetii* induced an M1 transcriptional program and higher TNF release by MDMs from females. After vaccination, the expression of inflammatory genes is higher in PBMCs from females than in those from males (25). In contrast, PBMCs from males release more TNF than those of females after LPS stimulation (26). It is likely that the M1 profile of MDMs from females contributed to their increased microbicidal activity against *C. burnetii*. This is consistent with the control of acute infections due to *Listeria monocytogenes* or *Salmonella typhi* by M1 macrophages (12). The protective role of TNF, a major cytokine of M1 macrophages, has been reported in *Mycobacterium tuberculosis*, *Candida albicans* and *Giardia lamblia* infections, in which it limits pathogen persistence and improves survival of mice (27–29). Conversely, *IL1RA* gene expression was increased in MDMs from males compared to those from females. IL-1RA reduces resistance to intracellular organisms such as *L. monocytogenes* or mycobacteria (30). Mice inactivated for the *IL1RA* gene are less infected by *L. monocytogenes* (31), and repeated injections of IL-1RA result in enhanced bacterial growth in *M. avium*-infected mice (32). We previously showed that IL1-RA was a marker of acute Q fever (33), and it should be investigated in future studies of sexual dimorphism.

Next, we wondered if the different responses of MDMs from males and females to *C. burnetii* are dependent on sex hormones. Estrogens are considered as inflammatory molecules, while testosterone as an anti-inflammatory hormone. Although the resistance of MDMs from females to *C. burnetii* infection seemed to be related to their M1 program, adding estradiol E2 did not significantly affect the expression of inflammatory cytokines. It is likely that estrogen impregnation of MDMs from females is sufficient and could not be amplified. These findings are distinct from other infections, in which estradiol E2 is clearly protective (34, 35). In fact, the activity of estradiol E2 on cytokine production is more complex than initially expected. Estradiol E2 enhances cell-mediated and humoral immune responses: low levels promote Th1 responses, while high concentrations augment Th2 responses (36). Pregnancy is a good example of the non-protective role of high levels of estrogens. During pregnancy, estradiol has anti-inflammatory effects, and susceptibility to *C. burnetii* infection is increased (2).

In contrast to the ambiguous effect of estrogens on the inflammatory response, testosterone clearly plays a regulatory role in immune response. We found that testosterone amplified immunoregulatory genes such as those encoding IL-10 and TGF-β in MDMs from males infected by *C. burnetii*. This enhancement was not restricted to gene expression, but also involved TGF-β release and was related to bacterial uptake, thus appearing as a biomarker of male susceptibility to *C. burnetii*. While the role of IL-10 in the persistence of *C. burnetii* in macrophages has been clearly described (37), that of TGF-β has been poorly investigated. We reported that TGF-β does not affect the persistence of *C. burnetii* in human monocytes (14), and that TGF-β production is higher in mononuclear cells from Q fever patients than in control cells (15), but unfortunately sex bias was not assessed in these two studies. Our results are consistent with most papers describing the immunoregulatory role of androgens. IL-10 production is higher in stimulated PBMCs from males than in those from females in an androgen concentration-dependent manner (38). Androgens also enhance IL-4-induced M2 polarization of murine bone marrow-derived macrophages and alveolar macrophages (39). In addition, the molecular species of androgens may differently impact the responses of myeloid cells. The expression of inflammatory cytokines by human monocytes and MDMs is reduced in the presence of testosterone, but dehydroepiandrosterone induces IL-6 and TNF production by human monocytes stimulated by LPS (24).

This study presents some limitations. First, the lack of investigation of sex bias in patients with Q fever. It only enables the assessment of sex influence on the functional properties of myeloid cells infected *in vitro* by *C. burnetii*. In addition, the immune response is largely dependent on other cell components, such as dendritic and T cells, which may be better biomarkers of sex dimorphism in Q fever. Indeed, the phenotypic evaluation of CD4^+^ T cells, CD8^+^ T cells, Tregs, NK cells, B cells and dendritic cells reveals variations in patients as compared with controls, but no significant differences between males and females with Q fever, whatever the clinical form (unpublished data). It is also noteworthy that the conditions explored here exclude those observed during pregnancy. Furthermore, the *in vitro* model used in this study shows some limitations. In this study we decided to use primary cells from males and females cultivated *in vitro* that oversimplifies the question of sex but constitute a limitation based on the *in vitro* environment. Indeed, we reported a large heterogeneity of donors including a disparity of the values and high standard deviations obtained. These observations highlight the necessity to conserve cells in an *in vivo* environments with hormones, metabolites and neural inputs that contribute to define two different male and female biological systems for cells (40). In addition, hormonal factors such as the reproductive status of an individual or environmental factors are important determinants of sex-related differences in immune responses which justify the multiplicity of animal models.

In conclusion, we have demonstrated that sex hormones can affect cellular responses to *C. burnetii* infection but not that it is necessarily dependent on sex hormones. These results open new perspectives for studying the susceptibility of males and resistance of females to *C. burnetii* infection. The role of sex hormones as an adjuvant in the personalized treatment of infections is a future challenge.

## Data Availability Statement

The original contributions presented in the study are included in the article/**Supplementary Material**. Further inquiries can be directed to the corresponding author.

## Ethics Statement

A convention No.7828 was established between our laboratory and the Etablissement Français du Sang (Marseille, France). The patients/participants provided their written informed consent to participate in this study.

## Author Contributions

LG, CM, AL, and SM realized experiment. LG, CM, BD, and SM analyzed the data. CM, DR, ML, SM, and J-LM supervised the project. LG, CM, SM, and J-LM wrote the manuscript. All authors contributed to the article and approved the submitted version.

## Funding

This work was supported by the French Government under the “investissement d’avenir” (“investments for the future”) program managed by the Agence Nationale de la Recherche (ANR, fr: National Agency for research), (reference: Méditerranée infection 10-IAHU-03).

## Conflict of Interest

The authors declare that the research was conducted in the absence of any commercial or financial relationships that could be construed as a potential conflict of interest.

## Publisher’s Note

All claims expressed in this article are solely those of the authors and do not necessarily represent those of their affiliated organizations, or those of the publisher, the editors and the reviewers. Any product that may be evaluated in this article, or claim that may be made by its manufacturer, is not guaranteed or endorsed by the publisher.

## Supplementary Material

Supplementary Figure 1 Experimental design. Monocytes from healthy donors (including males and females) were isolated from PBMCs after adhesion for 2 hours. Macrophages were obtained after monocytes differentiation for 7 days. **(A)** Monocytes and macrophages were infected by *C. burnetii* (50 bacteria per cell) or stimulated by 1 µg/mL LPS at different times according to the type of investigation. **(B)** In some experiments, MDMs from males and females were pre-treated for 24 hours with 1 nM estradiol E2 and 1 nM testosterone, respectively, before *C. burnetii* stimulation.

Supplementary Figure 2 Dose tests of sex hormones in MDM response to LPS. Monocytes from healthy donors (including males and females) were isolated from PBMCs after adhesion for 2 hours. Macrophages were obtained after monocytes differentiation for 7 days. MDMs from males (n=3) and females (n=3) were pre-treated for 24 hours with 0, 1, 10, 100 or 1000 nM estradiol E2 and testosterone, respectively, before LPS stimulation (1 µg/mL). The release of IL-10 and TFN-α by MDMs stimulated by LPS for 24 hours was determined by immunoassays. Data for represent mean ± standard error of the mean (duplicate was performed for each individual).

Supplementary Figure 3 C*. burnetii* uptake and persistence. **(A)** Monocytes and MDMs from the same healthy donors from males (n=3) and females (n=3) were infected with *C. burnetii* (50 bacteria per cell) for 4 hours. Each donor was characterized by a different symbol. The number of bacterial DNA copies within cells was determined by qPCR. After the bacterial uptake (designated day 0), **(B)** monocytes and **(C)** MDMs were cultivated for 9 days, and the presence of bacterial DNA copies was assessed every 3 days. The bacterial fate at day 3, 6 and 9 was calculated in percentage relative to day 0. Data for men (in grey) and female (in white) represent mean ± standard error of the mean (duplicate was performed for each individual). Statistical analyses were performed using Mann-Whitney *U* test (men *vs.* women).

Supplementary Figure 4 Effect of testosterone on the immunoregulatory response of LPS-stimulated MDMs. MDMs from males (n=8) were pre-treated with testosterone for 24 hours before a 6-hour stimulation by LPS. The expression of immunoregulatory genes was investigated by qRT-PCR and expressed as fold change relative to unstimulated cells was calculated. Gene expression was considered modulated when the fold change was ≥ 1.5 (indicated by the dotted line). Data for men (in grey), with (striped bar) or without pre-treatment, represent mean ± standard error of the mean (duplicate was performed for each individual). Statistical analyses were performed using Mann-Whitney *U* test (men *vs.* women). **p ≤* 0.05.

Supplementary Figure 5 Role of sex hormones in MDM responses to *C. burnetii.* MDMs from healthy donors from males (n=3) and females (n=3) were treated with testosterone or with estradiol E2 for 24 hours before *C. burnetii* (50 bacteria per cell) stimulation. The expression of MDM **(A)** M_1_ and **(B)** M_2_ polarization genes was investigated by qRT-PCR, constant amount of total DNA and expressed as fold change relative to unstimulated cells was calculated. Gene expression was considered modulated when the fold change was ≥ 1.5 (indicated by the dotted line). Data for men (in grey) and female (in white), with (striped bar) or without pre-treatment, represent mean ± standard error of the mean (duplicate was performed for each individual). Statistical analyses were performed using Mann-Whitney *U* test (men *vs.* women). **p ≤* 0.05.

Supplementary Table 1 List of primers for inflammatory (M1)/immunomodulatory (M2) genes

## References

[1] Eldin C Melenotte C Mediannikov O Ghigo E Million M Edouard S. From Q Fever to *Coxiella Burnetii* Infection: A Paradigm Change. Clin Microbiol Rev (2017) 30:115–90. doi: 10.1128/CMR.00045-16 PMC5217791 27856520

[2] Melenotte C Protopopescu C Million M Edouard S Carrieri MP Eldin C. Clinical Features and Complications of *Coxiella Burnetii* Infections From the French National Reference Center for Q Fever. JAMA Netw Open (2018) 1:e181580–e181580. doi: 10.1001/jamanetworkopen.2018.1580 30646123 PMC6324270

[3] Dupont H Raoult D Brouqui P Janbon F Peyramond D Weiller P-J. Epidemiologic Features and Clinical Presentation of Acute Q Fever in Hospitalized Patients: 323 French Cases. Am J Med (1992) 93:427–34. doi: 10.1016/0002-9343(92)90173-9 1415306

[4] Melenotte C Gay L Mezouar S Bardin N Raoult D Mège J-L. The Sexual Dimorphism of Anticardiolipin Autoantibodies in Acute Q Fever Patients. Clin Microbiol Infect (2019) 25:763. doi: 10.1016/j.cmi.2019.02.030 30898724

[5] Houpikian P Raoult D. Blood Culture-Negative Endocarditis in a Reference Center: Etiologic Diagnosis of 348 Cases. Med (Baltimore) (2005) 84:162–73. doi: 10.1097/01.md.0000165658.82869.17 15879906

[6] Mège J-L Bretelle F Leone M. Sex and Bacterial Infectious Diseases. N Microbes New Infect (2018) 26:S100–3. doi: 10.1016/j.nmni.2018.05.010 PMC6205577 30402251

[7] Marriott I Huet-Hudson YM. Sexual Dimorphism in Innate Immune Responses to Infectious Organisms. Immunol Res (2006) 34:177–92. doi: 10.1385/IR:34:3:177 16891670

[8] Maltezou HC. Raoult D. Q Fever in Children. Lancet Infect Dis (2002) 2:686–91. doi: 10.1016/S1473-3099(02)00440-1 12409049

[9] Howard ZP Omsland A. Selective Inhibition of *Coxiella Burnetii* Replication by the Steroid Hormone Progesterone. Infect Immun (2020) 88:e00894–19. doi: 10.1128/IAI.00894-19 PMC7671902 32928965

[10] Textoris J Ban LH Capo C Raoult D Leone M Mège J-L. Sex-Related Differences in Gene Expression Following *Coxiella Burnetii* Infection in Mice: Potential Role of Circadian Rhythm. PloS One (2010) 5:e12190. doi: 10.1371/journal.pone.0012190 20730052 PMC2921390

[11] Leone M Honstettre A Lepidi H Capo C Bayard F Raoult D. Effect of Sex on *Coxiella Burnetii* Infection: Protective Role of 17beta-Estradiol. J Infect Dis (2004) 189:339–45. doi: 10.1086/380798 14722900

[12] Benoit M Desnues B Mege J-L. Macrophage Polarization in Bacterial Infections. J Immunol (2008) 181:3733–9. doi: 10.4049/jimmunol.181.6.3733 18768823

[13] Benoit M Barbarat B Bernard A Olive D Mege J-L. *Coxiella Burnetii*, the Agent of Q Fever, Stimulates an Atypical M2 Activation Program in Human Macrophages. Eur J Immunol (2008) 38:1065–70. doi: 10.1002/eji.200738067 18350541

[14] Ghigo E Capo C Raoult D Mege JL. Interleukin-10 Stimulates *Coxiella Burnetii* Replication in Human Monocytes Through Tumor Necrosis Factor Down-Modulation: Role in Microbicidal Defect of Q Fever. Infect Immun (2001) 69:2345–52. doi: 10.1128/IAI.69.4.2345-2352.2001 PMC98164 11254592

[15] Capo C Zaffran Y Zugun F Houpikian P Raoult D Mege JL. Production of Interleukin-10 and Transforming Growth Factor Beta by Peripheral Blood Mononuclear Cells in Q Fever Endocarditis. Infect Immun (1996) 64:4143–7. doi: 10.1128/iai.64.10.4143-4147.1996 PMC174349 8926081

[16] Ben Amara A Bechah Y Mege J-L. “Immune Response and Coxiella Burnetii Invasion,”. In: Toman R Heinzen RA Samuel JE Mege J-L, editors. Coxiella Burnetii: Recent Advances and New Perspectives in Research of the Q Fever Bacterium Advances in Experimental Medicine and Biology. Springer Netherlands: Dordrecht (2012). p. 287–98. doi: 10.1007/978-94-007-4315-1_15 22711638

[17] Mezouar S Benammar I Boumaza A Diallo AB Chartier C Buffat C. Full-Term Human Placental Macrophages Eliminate *Coxiella Burnetii* Through an IFN-γ Autocrine Loop. Front Microbiol (2019) 10:2434. doi: 10.3389/fmicb.2019.02434 31749776 PMC6842979

[18] Ben Amara A Gorvel L Baulan K Derain-Court J Buffat C Vérollet C. Placental Macrophages are Impaired in Chorioamnionitis, an Infectious Pathology of the Placenta. J Immunol (2013) 191:5501–14. doi: 10.4049/jimmunol.1300988 24163411

[19] D’Agostino P Milano S Barbera C Di Bella G La Rosa M Ferlazzo V. Sex Hormones Modulate Inflammatory Mediators Produced by Macrophages. Ann N Y Acad Sci (1999) 876:426–9. doi: 10.1111/j.1749-6632.1999.tb07667.x 10415638

[20] Spitzer JA Zhang P. Gender Differences in Phagocytic Responses in the Blood and Liver, and the Generation of Cytokine-Induced Neutrophil Chemoattractant in the Liver of Acutely Ethanol-Intoxicated Rats. Alcohol Clin Exp Res (1996) 20:914–20. doi: 10.1111/j.1530-0277.1996.tb05271.x 8865968

[21] Scotland RS Stables MJ Madalli S Watson P Gilroy DW. Sex Differences in Resident Immune Cell Phenotype Underlie More Efficient Acute Inflammatory Responses in Female Mice. Blood (2011) 118:5918–27. doi: 10.1182/blood-2011-03-340281 PMC5363818 21911834

[22] Chao TC Phuangsab A Van Alten PJ Walter RJ. Steroid Sex Hormones and Macrophage Function: Regulation of Chemiluminescence and Phagocytosis. Am J Reprod Immunol N Y N 1989 (1996) 35:106–13. doi: 10.1111/j.1600-0897.1996.tb00015.x 8839138

[23] Schneider CP Schwacha MG Samy TSA Bland KI Chaudry IH. Androgen-Mediated Modulation of Macrophage Function After Trauma-Hemorrhage: Central Role of 5α-Dihydrotestosterone. J Appl Physiol (2003) 95:104–12. doi: 10.1152/japplphysiol.00182.2003 12665535

[24] Becerra-Diaz M Song M Heller N. Androgen and Androgen Receptors as Regulators of Monocyte and Macrophage Biology in the Healthy and Diseased Lung. Front Immunol (2020) 11:1698. doi: 10.3389/fimmu.2020.01698 32849595 PMC7426504

[25] Klein SL Jedlicka A Pekosz A. The Xs and Y of Immune Responses to Viral Vaccines. Lancet Infect Dis (2010) 10:338–49. doi: 10.1016/S1473-3099(10)70049-9 PMC6467501 20417416

[26] Klein SL Flanagan KL. Sex Differences in Immune Responses. Nat Rev Immunol (2016) 16:626–38. doi: 10.1038/nri.2016.90 27546235

[27] Flynn JL Goldstein MM Chan J Triebold KJ Pfeffer K Lowenstein CJ. Tumor Necrosis Factor-α Is Required in the Protective Immune Response Against Mycobacterium Tuberculosis in Mice. Immunity (1995) 2:561–72. doi: 10.1016/1074-7613(95)90001-2 7540941

[28] Louie A Baltch AL Smith RP Franke MA Ritz WJ Singh JK. Tumor Necrosis Factor Alpha has a Protective Role in a Murine Model of Systemic Candidiasis. Infect Immun (1994) 62:2761–72. doi: 10.1128/iai.62.7.2761-2772.1994 PMC302879 8005666

[29] Zhou P Li E Shea-Donohue T Singer SM. Tumour Necrosis Factor α Contributes to Protection Against *Giardia Lamblia* Infection in Mice. Parasite Immunol (2007) 29:367–74. doi: 10.1111/j.1365-3024.2007.00953.x PMC2443547 17576366

[30] Arend WP Malyak M Guthridge CJ Gabay C. Interleukin-1 Receptor Antagonist: Role in Biology. Annu Rev Immunol (1998) 16:27–55. doi: 10.1146/annurev.immunol.16.1.27 9597123

[31] Hirsch E Irikura VM Paul SM Hirsh D. Functions of Interleukin 1 Receptor Antagonist in Gene Knockout and Overproducing Mice. Proc Natl Acad Sci USA (1996) 93:11008–13. doi: 10.1073/pnas.93.20.11008 PMC38274 8855299

[32] Denis M Ghadirian E. Interleukin-1 Is Involved in Mouse Resistance to. Mycobacterium Avium Infect Immun (1994) 62:457–61. doi: 10.1128/iai.62.2.457-461.1994 PMC186129 8300206

[33] Capo C Amirayan N Ghigo E Raoult D Mege J-L. Circulating Cytokine Balance and Activation Markers of Leucocytes in Q Fever. Clin Exp Immunol (1999) 115:120. doi: 10.1046/j.1365-2249.1999.00786.x 9933430 PMC1905180

[34] Saia RS Garcia FM Cárnio EC. Estradiol Protects Female Rats Against Sepsis Induced by *Enterococcus Faecalis* Improving Leukocyte Bactericidal Activity. Steroids (2015) 102:17–26. doi: 10.1016/j.steroids.2015.06.016 26143494

[35] Merkel SM Alexander S Zufall E Oliver JD Huet-Hudson YM. Essential Role for Estrogen in Protection Against *Vibrio Vulnificus*-Induced Endotoxic Shock. Infect Immun (2001) 69:6119–22. doi: 10.1128/IAI.69.10.6119-6122.2001 PMC98741 11553550

[36] Bouman A Heineman MJ Faas MM. Sex Hormones and the Immune Response in Humans. Hum Reprod Update (2005) 11:411–23. doi: 10.1093/humupd/dmi008 15817524

[37] Meghari S Bechah Y Capo C Lepidi H Raoult D Murray PJ. Persistent *Coxiella Burnetii* Infection in Mice Overexpressing IL-10: An Efficient Model for Chronic Q Fever Pathogenesis. PloS Pathog (2008) 4:e23. doi: 10.1371/journal.ppat.0040023 18248094 PMC2222951

[38] Torcia MG Nencioni L Clemente AM Civitelli L Celestino I Limongi D. Sex Differences in the Response to Viral Infections: TLR8 and TLR9 Ligand Stimulation Induce Higher IL10 Production in Males. PloS One (2012) 7:e39853. doi: 10.1371/journal.pone.0039853 22768144 PMC3387221

[39] Becerra-Diaz M Strickland AB Keselman A Heller NM. Androgen and Androgen Receptor as Enhancers of M2 Macrophage Polarization in Allergic Lung Inflammation. J Immunol (2018) 201:2923–33. doi: 10.4049/jimmunol.1800352 PMC6219904 30305328

[40] Mauvais-Jarvis F Arnold AP. Reue K. A Guide for the Design of Pre-Clinical Studies on Sex Differences in Metabolism. Cell Metab (2017) 25:1216–30. doi: 10.1016/j.cmet.2017.04.033 PMC5516948 28591630

