# Supplementary figures and images for: Impact of Sex Hormones on Macrophage Responses to Coxiella burnetii
Source: Front Immunol. 2021 Dec 20;12:705088. doi: 10.3389/fimmu.2021.705088 (PMC8720845; doi:10.3389/fimmu.2021.705088)

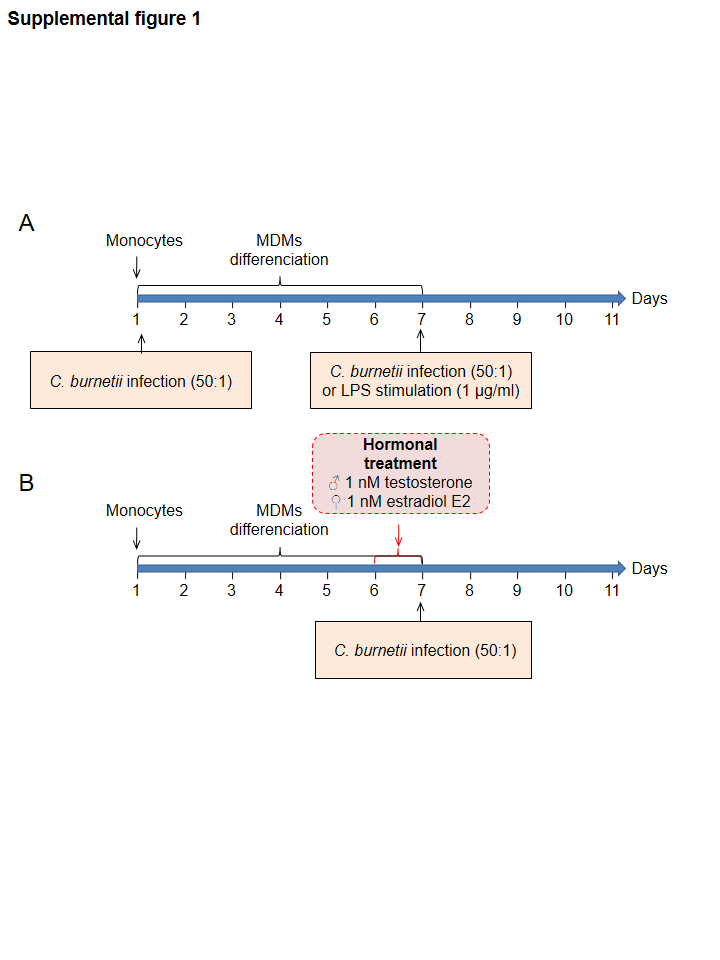

Supplement: Supplementary Figure 1 — Experimental design. Monocytes from healthy donors (including males and females) were isolated from PBMCs after adhesion for 2 hours. Macrophages were obtained after monocytes differentiation for 7 days. (A) Monocytes and macrophages were infected by C. burnetii (50 bacteria per cell) or stimulated by 1 µg/mL LPS at different times according to the type of investigation. (B) In some experiments, MDMs from males and females were pre-treated for 24 hours with 1 nM estradiol E2 and 1 nM testosterone, respectively, before C. burnetii stimulation. [file Image_1.tif]

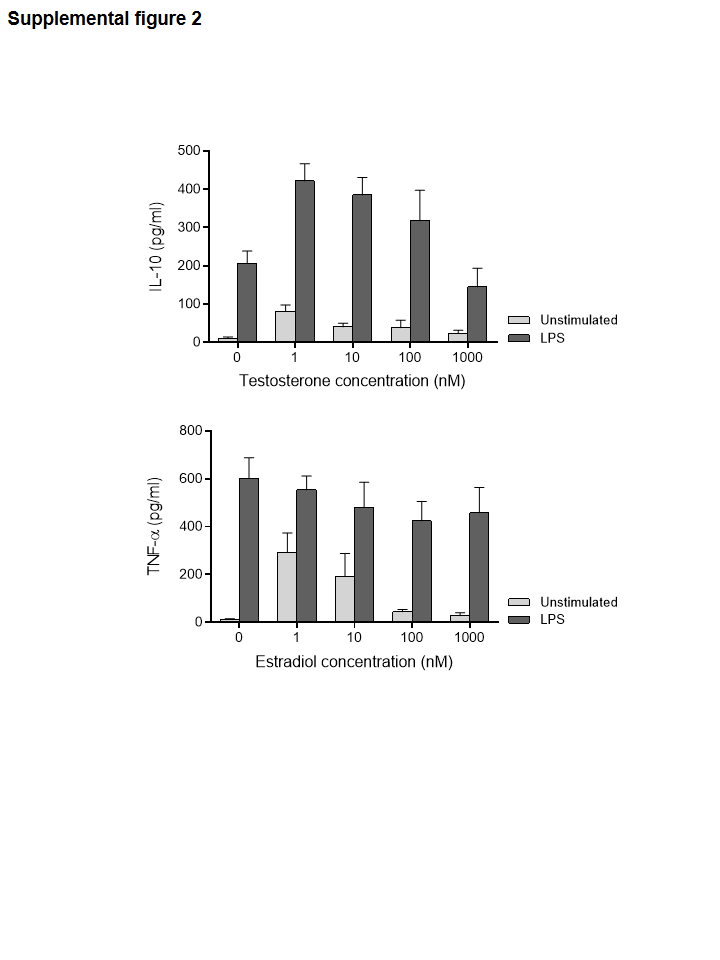

Supplement: Supplementary Figure 2 — Dose tests of sex hormones in MDM response to LPS. Monocytes from healthy donors (including males and females) were isolated from PBMCs after adhesion for 2 hours. Macrophages were obtained after monocytes differentiation for 7 days. MDMs from males (n=3) and females (n=3) were pre-treated for 24 hours with 0, 1, 10, 100 or 1000 nM estradiol E2 and testosterone, respectively, before LPS stimulation (1 µg/mL). The release of IL-10 and TFN-α by MDMs stimulated by LPS for 24 hours was determined by immunoassays. Data for represent mean ± standard error of the mean (duplicate was performed for each individual). [file Image_2.tif]

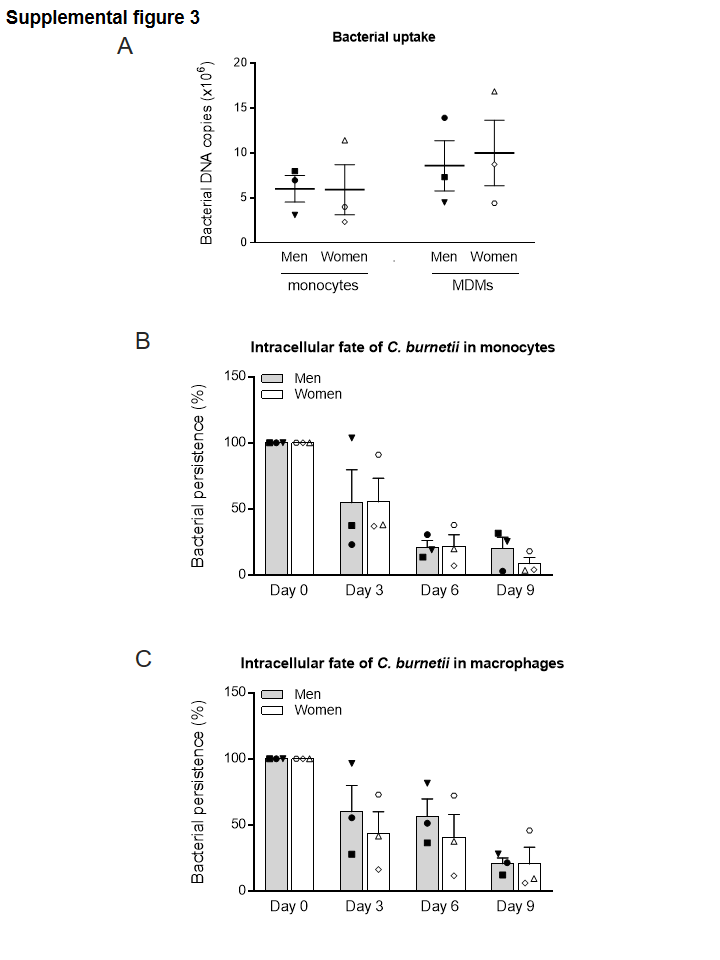

Supplement: Supplementary Figure 3 — C. burnetii uptake and persistence. (A) Monocytes and MDMs from the same healthy donors from males (n=3) and females (n=3) were infected with C. burnetii (50 bacteria per cell) for 4 hours. Each donor was characterized by a different symbol. The number of bacterial DNA copies within cells was determined by qPCR. After the bacterial uptake (designated day 0), (B) monocytes and (C) MDMs were cultivated for 9 days, and the presence of bacterial DNA copies was assessed every 3 days. The bacterial fate at day 3, 6 and 9 was calculated in percentage relative to day 0. Data for men (in grey) and female (in white) represent mean ± standard error of the mean (duplicate was performed for each individual). Statistical analyses were performed using Mann-Whitney U test (men vs. women). [file Image_3.tif]

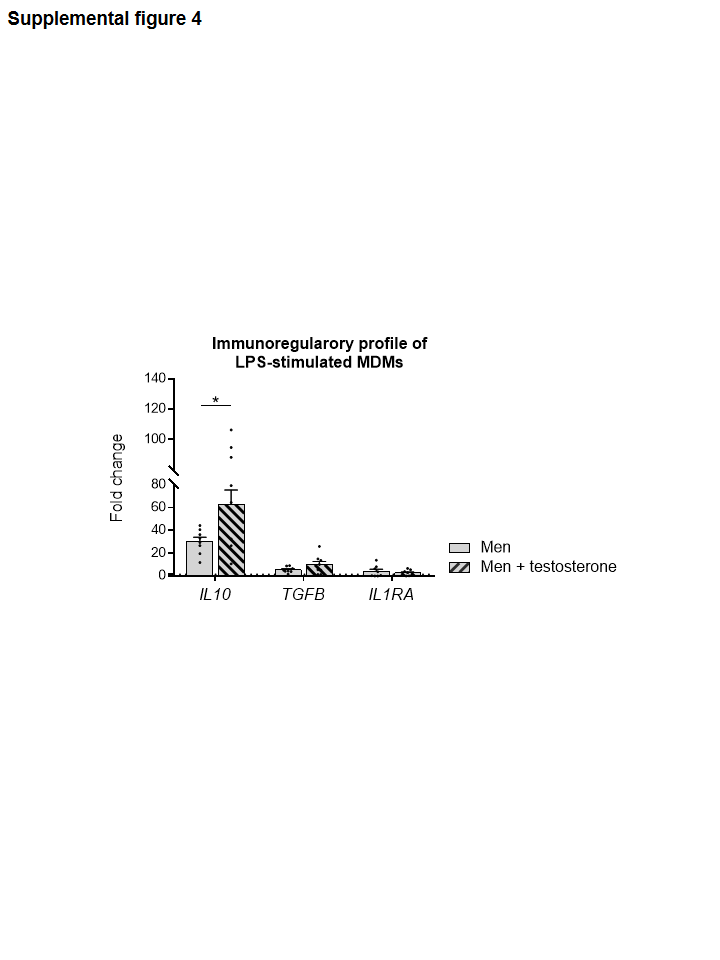

Supplement: Supplementary Figure 4 — Effect of testosterone on the immunoregulatory response of LPS-stimulated MDMs. MDMs from males (n=8) were pre-treated with testosterone for 24 hours before a 6-hour stimulation by LPS. The expression of immunoregulatory genes was investigated by qRT-PCR and expressed as fold change relative to unstimulated cells was calculated. Gene expression was considered modulated when the fold change was ≥ 1.5 (indicated by the dotted line). Data for men (in grey), with (striped bar) or without pre-treatment, represent mean ± standard error of the mean (duplicate was performed for each individual). Statistical analyses were performed using Mann-Whitney U test (men vs. women). *p ≤ 0.05. [file Image_4.tif]

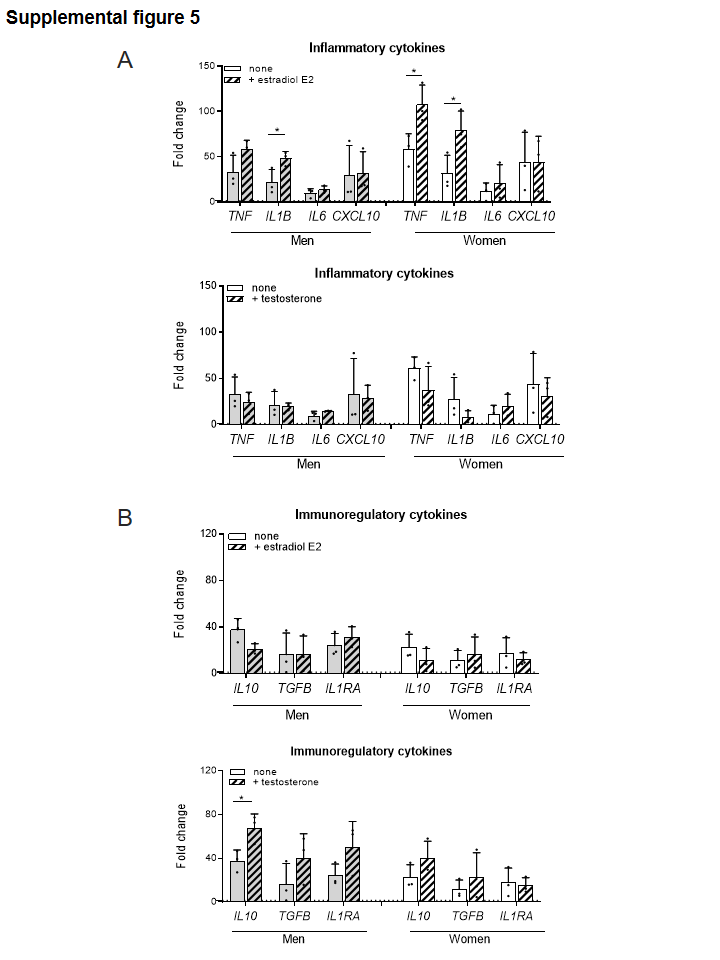

Supplement: Supplementary Figure 5 — Role of sex hormones in MDM responses to C. burnetii. MDMs from healthy donors from males (n=3) and females (n=3) were treated with testosterone or with estradiol E2 for 24 hours before C. burnetii (50 bacteria per cell) stimulation. The expression of MDM (A) M1 and (B) M2 polarization genes was investigated by qRT-PCR, constant amount of total DNA and expressed as fold change relative to unstimulated cells was calculated. Gene expression was considered modulated when the fold change was ≥ 1.5 (indicated by the dotted line). Data for men (in grey) and female (in white), with (striped bar) or without pre-treatment, represent mean ± standard error of the mean (duplicate was performed for each individual). Statistical analyses were performed using Mann-Whitney U test (men vs. women). *p ≤ 0.05. [file Image_5.tif]
