# Supplementary material for: Impact of Sex Hormones on Macrophage Responses to Coxiella burnetii
Source: Front Immunol. 2021 Dec 20;12:705088. doi: 10.3389/fimmu.2021.705088 (PMC8720845; doi:10.3389/fimmu.2021.705088)
Supplement: Supplementary Table 1 — List of primers for inflammatory (M1)/immunomodulatory (M2) genes [file Table_1.pdf]

Supplementary table 1. List of primers for inflammatory (M1)/ immunomodulatory (M2) genes

Gene symbol	Forward primer (5'-3')	Reverse primer (5'-3')
ACTB	GGAAATCGTGCGTGACATTA	AGGAGGAAGGCTGGAAGAG
	**M1 genes**	
IL1B	CAGCACCTCTCAAGCAGAAAAC	GTTGGGCATTGGTGTAGACAAC
IL6	CCAGGAGAAGATTCCAAAGATG	GGAAGGTTCAGGTTGTTTTCTG
IL7	GACAGCATGAAAGAAATTGGTAGC	CAACTTGCGAGCAGCACGGAAT
IL12P35	GTCACTGCCCGAATTCTGAAAG	TGCAAAGCTTCTGATGGATCCT
IL23A	AATAATCCTCCCCAAACTGTCC	CAGCAACCCTGAGTCCCTAA
TNF	AGGAGAAGAGGCTGAGGAACAAG	GAGGGAGAGAAGCAACTACAGACC
CCR7	GAGGGGATGAGTGTGCTTTTAG	GACCAGAGATAGTGGGGAGACTT
CXCL10	GGAAATCGTGCGTGACATTA	AGGAAGGAAGGCTGGAAGAG
	**M2 genes**	
IL10	GGGGGTTGAGGTATCAGAGGTAA	GCTCCAAGAGAAAGGCATCTACA
TGFB	GACATCAAAAGATAACCACTC	TCTATGACAAGTTCAAGCAGA
CCL16	TCTCTCCTTGTCCTCATCCTTATC	GACTTCTCGGTTCCTCTTGGT
IL1RA	TCTATCACCAGACTTGACACA	CCTAATCACTCTCCTCCTCTTCC
IL13RA	TAGATGGCCATGAAGAGGATGC	CCAAGACCTAGGGATCACAACC
